# Virtual Reality in the Management of Chronic Low Back Pain: A Scoping Review

**DOI:** 10.3389/fpain.2022.856935

**Published:** 2022-03-07

**Authors:** Ameet S. Nagpal, Aditya Raghunandan, Faye Tata, Daniel Kibler, Donald McGeary

**Affiliations:** ^1^Department of Anesthesiology, UT Health San Antonio, San Antonio, TX, United States; ^2^Department of Rehabilitation Medicine, UT Health San Antonio, San Antonio, TX, United States; ^3^Department of Psychiatry, UT Health San Antonio, San Antonio, TX, United States

**Keywords:** chronic low back pain, virtual reality, augmented reality, low back pain, scoping review

## Abstract

Virtual reality (VR) is a burgeoning treatment option for chronic pain. Its use has been heterogenous in the literature. This scoping review assesses the current literature for the use of VR in the treatment of chronic low back pain (CLBP). The following themes were identified by the analysis: safety and feasibility of VR, quality of life associated with VR treatment for CLBP, efficacy of VR to treat CLBP, and efficacy of VR to treat functional changes associated with CLBP. Gaps were identified after analysis of the extant literature. Although the nascent research uncovered in this scoping review found good evidence for safety and tolerability of VR, more studies of safety, acceptance, and satisfaction are recommended including focused studies of spinal pain risks specific to use of VR. Overall, the methodological quality of studies reviewed in this scoping review was poor and outcomes were limited to short-term posttreatment outcomes.

## Introduction

Non-specific low back pain (NSLBP) is defined as low back pain not attributable to a known cause, whereas chronic low back pain (CLBP) refers to a myriad of potential etiologies which cause ongoing pain for longer than 12 weeks (1–3). In the US, the prevalence of chronic low back pain in the adult population (over the age of 18) ranges between 10 and 30% with a lifetime prevalence as high as 65–80% (4). These high prevalence rates correspond to an associated significant socioeconomic burden in the United States (US). A retrospective study performed by Mehra et al. analyzed nationwide integrated medical/pharmaceutical claims data and revealed that the total direct costs of CLBP-related resource use were ~$96 million over a 12-month follow-up for all CLBP patients, with a mean annual cost of $2,426 per patient (5).

Given this high burden of care, it is prudent to provide the most cost-effective and evidence-based diagnosis and treatment options to patients with NSLBP and CLBP. The recent North American Spine Society (NASS) 2020 Clinical Guideline for the Diagnosis and Treatment of Low Back Pain addresses a variety of evidence-based recommendations for the management of non-specific low back pain. Providing an accurate diagnosis for the etiology of CLBP can clarify management recommendations, however, this may not be possible in patients where non-structural causes of low back pain may be considered. A recommended multimodal non-interventional approach to management of CLBP includes: cognitive behavioral therapy, patient education, treatments targeting fear avoidance, McKenzie exercise [directional preference, centralization and mechanical diagnosis and therapy (MDT)], yoga, aerobic exercise, work hardening or conditioning, non-selective NSAIDs, judicious use of opioid medications. Interventional management options include radiofrequency neurotomy of medial branches of the lumbar spine for facet-mediated pain, intra-articular joint injections or lateral branch neurotomy for sacroiliac joint pain, and several experimental procedures for discogenic pain (6). For patients who do not improve with these measures, surgical management may be an option.

In addition to the non-surgical options detailed above, extended reality (XR) technologies provide a new alternative to medication and interventions for management of CLBP. XR is an umbrella term that describes emerging immersive technologies that can merge the physical and virtual environments. Some of the common immersive technologies used for pain management include virtual reality (VR), augmented reality (AR), and mixed reality (MR) (7). VR was adopted early by the gaming and entertainment industry to provide a fully immersive experience into a simulated digital environment. Individuals must wear a VR head-mounted display to obtain an immersive feel for an artificial world. VR has been shown as an effective treatment option for managing acute pain, but the body of research for chronic pain is still in the early stages (8). In AR, however, virtual information and/or objects can be overlaid on the real world. This virtual information can enhance the physical world with digital details such as images, text, and animation. These virtual details can be delivered via glasses, screens, tablets, and smartphones. AR has been used as a safe way to help trainees practice routine and complex spine surgical techniques (9). Finally, in MR virtual and physical objects co-exist and can interact with one another in real-time. This is the latest immersive technology and alternatively referred to as hybrid reality for which a specific MR headset is required. The scope for this technology is vast with applications in medical education training, research, communication, and clinical treatment (10).

Virtual reality offers novel immersion to pain rehabilitation that may enhance outcomes from other rehabilitation protocols (as an adjunctive technology) and provide direct analgesic effects through yet unspecified mechanisms (11). However, the body of research examining VR for CLBP is nascent and early systematic reviews found little evidence supporting incremental benefit of physical rehabilitation with adjunctive VR (12). Research on VR for pain rehabilitation is rapidly growing, so this scoping review will specifically address the latest available evidence for VR as it relates to management of CLBP, identify and analyze current knowledge gaps, and serve as precursor to future systematic reviews.

## Methods

### Design

To understand the available range of extant research on the topic of VR as a treatment for CLBP, this scoping review followed the required 5 steps of a previously published framework (13). The five steps followed included:

1. Identification of the research question (“what does existing data demonstrate on the topic of utilizing VR for the treatment of CLBP”)
2. Identification of relevant studies
3. Study selection
4. Charting of data
5. Collating and summarizing and reporting findings

After following this framework, our team analyzed available literature in an attempt to develop themes, describe the current state of the literature, and identify research gaps.

### Search Strategy

Medline and Scopus were searched by members of the research team (ASN, AR, FT, and DK) utilizing the search string of (“virtual reality” AND “chronic low back pain”) with filters applied for human studies (no search suffixes used). These searches were performed at various intervals in July, August, September, and October of 2021. Forty-eight articles were initially identified by this strategy. Thirty articles were then excluded (11 were duplicates, 24 were excluded because they did not meet inclusion criteria), and so 13 total studies were included (14–26).

### Inclusion Criteria

Studies were included if VR was utilized for a study population with chronic pain primarily of the low back. English and non-English language studies were acceptable. Randomized controlled trials, prospective and retrospective cohort studies, and observational studies were included. Case reports and ongoing studies were excluded.

### Development of Themes

After the final included manuscripts were identified, each team member was responsible for reading each of these manuscripts. The team then met asynchronously on 3 different occasions to identify grouped themes associated with the published literature on the topic of VR and CLBP. A final asynchronous meeting occurred in order to identify literature gaps. There were no disagreements and therefore moderation for resolution was not necessary.

## Results

The following themes were identified by the analysis: safety and feasibility of VR, quality of life associated with VR treatment for CLBP, efficacy of VR to treat CLBP, and efficacy of VR to treat functional changes associated with CLBP. A summary analysis of all included articles to assess if the articles included a clinical trial, randomized, included a control, and justification for sample size was completed (Table 1). Although all included articles were clinical trials, two were single armed studies, one study acting as a proof-of-concept and the other to further validate its specific VR program. Most (eight out of thirteen) articles justified their chosen sample size ranging from 13 participants (14), to 179 participants (21). On average, sample size was approximately 50.

**Table 1:** Summary analysis of all included articles to assess for study rigor.

**1st Author**	**Reference #**	**Clinical trial?**	**Randomized?**	**Controlled?**	**Sample size justification?**
Hennessy	(14)	Yes	No	No	No
Thomas	(15)	Yes	Yes	Yes	Yes
Nambi	(16)	Yes	Yes	Yes	Yes
Nambi	(17)	Yes	Yes	Yes	Yes
Park	(18)	Yes	Yes	Yes	No
Karahan	(19)	Yes	Yes	Yes	No
Alemanno	(20)	Yes	No	No	Yes
Garcia	(21)	Yes	Yes	Yes	Yes
Matheve	(22)	Yes	Yes	Yes	Yes
Yilmaz	(23)	Yes	Yes	Yes	Yes
Kim	(24)	Yes	Yes	Yes	No
Yoo	(25)	Yes	Yes	Yes	No
Nambi	(26)	Yes	Yes	Yes	Yes

### Safety and Feasibility of VR

Four different studies employed unique scales and markers to observe the fundamental feasibility and safety of VR.

Hennessey et al. used the System Usability Scale (SUS), a 10-item survey specifically focusing on usability of a VR app, and the Treatment Evaluation Inventory (TEI), a 9-item questionnaire regarding acceptability of this VR program. Scores greater than or equal to the cut-off TEI score were observed in 92% of participants and 75% of participants scored above average for SUS, demonstrating the usability, or feasibility of this app (14).

In Thomas et al.'s study, participants rated their overall impressions of the VR game using a survey that was adapted from an existing measure of online health game acceptability, the Game Experience Survey. The Roland-Morris Disability Questionnaire (RMDQ) and McGill Pain Questionnaire (MPQ) were utilized, along with participant retention rate and use of pain medications, to examine safety and feasibility of their dodgeball VR program. The Game Experience Survey ratings suggested a high level of acceptability for the game with strong ratings of enjoyment combined with willingness to continue to play the game and recommend it to others with back pain. The RMPQ and MPQ demonstrated that regardless of study arm, participants did not have a change in physical disability over time and pain intensity did not increase. Only one participant took pain medication for back pain preemptively prior to a VR session and 100% retention of participants was observed (15).

Nambi et al. studied VR vs. isokinetic training in football players with CLBP, all groups showed significant improvements in all observed stress hormone levels, with exception of glucose and insulin, after 4 weeks of intervention. Insulin resistance, growth hormone, prolactin, ACTH, and cortisol showed significant improvements all around at 6 months follow up, although greater tendency toward improvement in the VR group was noted per the *post hoc* Bonferroni test (16). Likewise, when Nambi's team studied soccer players with CLBP, there were significant improvements in all stress hormones without HOMA-IR in all studied groups at 4 weeks. Significant improvements in glucose, prolactin, ACTH, and cortisol were observed in all groups at 6 months, although more so in the VR group (26).

### Quality of Life Associated With VR Treatment for CLBP

Four articles evaluated the impact of virtual reality on the participants' quality of life (QOL).

Nambi et al. used a player wellness questionnaire at baseline, at 4 weeks, 8 weeks, and 6 months of VR that utilizes a five-point Likert scale to assess fatigue, sleep quality, muscle soreness, stress, and mood. A *post hoc* analysis, and percentage of improvement between groups were statistically significant for the VR group showing improvement in wellness/quality of life (17).

Park assessed quality of life using the short form health survey (SF36). The SF36 is a 36-item participant reported survey, and within the 36 items there are two categories, physical health, and mental health. Aspects in the physical health category are physical functioning, role limits from physical health, pain, and general health perception. Mental health categories address emotional well-being, role limits due to mental health, social functioning, and energy level/fatigue. The Nintendo Wii (VR) group improved significantly in terms of role limitations due to mental health, energy/fatigue, and emotional well-being (18).

Karahan et al. measured QOL with the ankylosing spondylitis quality of life questionnaire. This is an eighteen-item disease specific questionnaire designed to address disease impact on aspects of life such as sleep, mood, motivation, coping, daily activities, independence, relationships, and social life. After the eight-week intervention the ASQOL scores improved significantly in the virtual reality group (19).

Alemanno et al. used the SF36 and the Beck Depression Inventory (BDI-II) to assess QOL. The The single arm treated with VR showed significant improvements in five of the eight subscale scores within the SF36: physical functioning, physical role functioning, bodily pain, vitality, and social role functioning. A significant improvement was seen in post treatment BDI-II scores (20).

### Efficacy of VR to Treat Chronic Low Back Pain (CLBP)

Ten studies studied the efficacy of virtual reality to treat CLBP. See Table 2 for further details.

**Table 2:** Efficacy of VR to treat pain (top portion of Table) and functional change (bottom of portion of Table) associated with CLBP.

**Theme: Efficacy of virtual reality to treat chronic low back pain**				
Article title	First author	Study type	Measurement tool	Summary
An 8-Week Self-Administered At-Home Behavioral Skills-Based Virtual Reality Program for Chronic Low Back Pain: Double-Blind, Randomized, Placebo-Controlled Trial Conducted During COVID-19	Garcia	Randomized control trial (RCT)	DVPRS, DVPRSII, PGIC, PCS, PSEQ2, CPAQ-8	When compared to sham VR, the VR group showed significantly lower average DVPRS scores and significantly higher PGIC scores. No significant differences between these groups were observed for pain coping symptoms.
Virtual reality distraction induces hypoalgesia in patients with chronic low back pain: a randomized controlled trial	Matheve	RCT	NPRS, PCS	There was significant reduction in pain intensity during and after exercise for the VR group compared to the control.
Comparative effects of isokinetic training and virtual reality training on sports performances in university football players with chronic low back pain-randomized controlled study	Nambi	RCT	VAS	The virtual reality group had a significant improvement in VAS pain scores.
Is physiotherapy integrated virtual walking effective on pain, function, and kinesiophobia in patients with non-specific low-back pain? Randomized controlled trial	Yilmaz	RCT	VAS	After treatment the VR group showed a significant improvement in VAS pain scores.
The effects of VR-based Wii Fit yoga on physical function in middle-aged female LBP patients	Kim	RCT	VAS	The virtual reality group had a significant improvement in VAS pain scores.
The effect of horse simulator riding on visual analogue scale, body composition and trunk strength in the patients with chronic low back pain	Yoo	RCT	VAS	The virtual reality group had a significant improvement in VAS pain scores.
The effectiveness of exergames in patients with ankylosing spondylitis: a randomized controlled trial	Karahan	RCT	VAS	The virtual reality group had a significant improvement in VAS pain scores.
The effects of the Nintendo Wii exercise program on chronic work-related low back pain in industrial workers	Park	RCT	VAS	The Lumbar stabilizing exercise group and VR group showed a significant improvement in VAS pain scores when compared to the control group.
Virtual reality or isokinetic training; its effect on pain, kinesiophobia and serum stress hormones in chronic low back pain: A randomized controlled trial	Nambi	RCT	VAS	*Post hoc* Bonferroni test showed improvement in pain intensity in the VRT and IKT group in comparison to the control group. Pain intensity decreased significantly between all groups and significant difference after 4 weeks and 6 months of follow up.
Short-Term Psychological and Hormonal Effects of Virtual Reality Training on Chronic Low Back Pain in Soccer Players	Nambi	RCT	VAS	Significant improvement in pain intensity in VR group compared to control and combined physical rehabilitation groups at 4 weeks and 6 months.
**Theme: Efficacy of virtual reality to treat functional change associated with chronic low back pain**
Feasibility and Safety of a Virtual Reality Dodgeball Intervention for Chronic Low Back Pain: A Randomized Clinical Trial	Thomas	RCT	Lumbar flexion excursion derived from Euler angles	No significant effects of group (game vs. control) on changes in lumbar spine flexion outside of gameplay.
Efficacy of virtual reality to reduce chronic low back pain: Proof-of-concept of a non-pharmacological approach on pain, quality of life, neuropsychological and functional outcome	Alemanno	Case Control	RMDQ, truncal ROM derived from Euler angles, a Repetition Index derived for proprioception	Significant improvement in participation, trunk functionality, average range of motion, and proprioception at end of study compared to start.
Is physiotherapy integrated virtual walking effective on pain, function, and kinesiophobia in patients with non-specific low-back pain? Randomised controlled trial	Yilmaz	RCT	ODI, TUG, 6MWT, single-leg balance test	After treatment, there was a significant difference in the TUG and 6MWT scores between control and experimental groups. No significant difference in ODI or balance tests between groups, but there was significant difference between pre and post treatments in each group.
The effects of VR-based Wii Fit yoga on physical function in middle-aged female LBP patients	Kim	RCT	ODI and RMDQ	Significant improvements between pre and post training ODI and RMDQ scores. Significant differences between ODI scores in groups and significant differences in ODI and RMDQ with regard to effect of time-by-group interaction.
The effect of horse simulator riding on visual analogue scale, body composition and trunk strength in the patients with chronic low back pain	Yoo	RCT	Total work and isokinetic torque for trunk flexion and extension measured with an isokinetic dynamometer	After 8 weeks, peak torque and total work were significantly enhanced only in the horse simulator riding group, and decreased in control group. Related ratios correlated.
The effectiveness of exergames in patients with ankylosing spondylitis: a randomized controlled trial	Karahan	RCT	BASFI	In exergame group, BASFI scores improved significantly after 8 weeks of the program and remained unchanged in control group. Intergroup comparison after 8th week showed significant improvement in exergame group BASFI scores.
The effects of the Nintendo Wii exercise program on chronic work-related low back pain in industrial workers	Park	RCT	Maximum isometric lifting weight, One legged stand test	All groups demonstrated a significant increase in back strength and the Nintendo Wii (VR) group was the only group that did not show a significant improvement in balance.
Comparative effects of isokinetic training and virtual reality training on sports performances in university football players with chronic low back pain-randomized controlled study	Nambi	RCT	40 m sprint, 4 × 5 sprint, submaximal shuttle run, Vertical jump, Countermovement jump, Squat jump	Significant improvement for virtual reality group and IKT group for the 40 m sprint, 4 × 5 sprint, and submaximal shuttle run, CJ and SJ. A *post hoc* analysis showed more tendencies of improvement in the VRT group compared to IKT.

At three points during Garcia et al.'s study, pre-intervention, during, and post intervention, average pain intensity was measured using the Defense and Veterans Pain Rating Scale (DVPRS). DVPRS uses a ten-point Likert scale to address how pain affects activity, sleep, mood, and stress. Pain improvement was measured with the Patient Global Impression of Change Scale (PGIC). By the end of the study, the VR group had significantly lower average DVPRS scores than the sham VR group and significant improvement in PGIC compared to the sham VR group. No significant differences between groups were observed for pain catastrophizing, pain self-efficacy, and pain acceptance (21).

Matheve et al. measured pain using the numeric pain rating scale (NPRS) and PCS. This study noted significant reduction in pain intensity during and after exercise for the VR group compared to the control. No significant changes were seen with pain catastrophizing and baseline pain intensity (22).

Multiple studies, including Yilmaz, Kim, Yoo, Karahan, Park and the three articles by Nambi used the visual analog scale (VAS) to measure VR efficacy to treat CLBP. Each study showed a significant improvement in VAS pain scores for the VRT group compared to the control group (16–19, 23–26). In Park et al.'s study, lumbar stabilizing exercise group and VR group both showed a significant reduction in VAS pain scores when compared to the control group (18). In Nambi's article that compared Virtual reality vs. isokinetic training to assess its effect on pain, kinesiophobia, and serum hormones, the *post hoc* Bonferroni test and graphical representation showed improvement in pain intensity in the VRT and IKT group in comparison to the control group (16).

### Efficacy of VR to Treat Changes in Function Associated With CLBP

Eight studies assessed the ability of VR to treat various components of functional changes associated with CLBP. See Table 2 for further details.

Outside of VR gameplay, Thomas et al. observed no significant effects with group (game vs. control) on change in lumbar flexion from initial standing posture to target contact (extracted with custom software) (15).

Alemanno et al. looked at kinematic data using a tracking system and measured the maximal and average truncal range of motion and an index of proprioception ranging from 0 to 1, where 1 corresponded to the maximal accuracy in trunk rotation. The statistical analyses of RMDQ and these kinematics data revealed a significant improvement in participation, trunk functionality, average range of motion, and proprioception after VR intervention (20).

Yoo et al. used an isokinetic dynamometer for measurements of force generated by the trunk. During trunk extension and flexion, peak torque (or rotational force) and total work were measured at specific angular speeds (30 degrees per second and 90 degrees per second, respectively). This data was used along with body weight, fat mass, and muscle mass to compute additional data to relate force measurements to a subject's body composition. After 8 weeks, peak torque and total work were significantly enhanced only in the horse simulator riding group and decreased in control group. Mentioned related ratios correlated (25).

In Yilmaz et al.'s study, participants of each group were evaluated for disability with the Oswestry Disability Index (ODI), and functional capacity with the Timed Up (TUG) and Go test plus 6-Minute Walk Test (6MWT). Balance ability was assessed with the Single-leg balance test. After treatment, there was a significant difference in the TUG and 6MWT scores between control and VR groups. There was no significant difference in ODI or balance tests between groups, but there was significant difference before and after treatments in each group (23).

Likewise, Kim et al. utilized ODI and RMDQ to examine functional level during pain and severity of disability with significant improvements between groups in pre and post training ODI scores. This study found significant differences in ODI and RMDQ scores between groups regarding effect of time-by-group interaction (24).

Before and after 8 weeks of intervention, Park et al.'s participants were evaluated for back strength and balance ability using the maximum isometric lifting weight and how long a subject could stand on one leg (One-legged Stand Test). All groups demonstrated a significant increase in back strength and the Nintendo Wii (VR) group was the only group that did not show a significant improvement in balance (18).

Specifically looking at sports performance in the setting of CLBP, Nambi et al. measured athlete agility before during and after intervention using the 40 m sprint, 4 × 5 sprint, submaximal shuttle run, vertical jump, countermovement jump, and squat jump. There was significant improvement for both the virtual reality group and the isokinetic training group for the 40 m sprint, 4 × 5 sprint, submaximal shuttle run, countermovement jump, and squat jump. A *post hoc* analysis showed more tendencies of improvement in the VRT group compared to Isokinetic Training (IKT) (17).

Karahan et al. were uniquely interested in Bath Ankylosing Spondylitis Functional Index (BAFSI) scores in their population. These improved significantly after 8 weeks of the VR program and remained unchanged in control group. Intergroup comparison after the 8th week showed significant improvement in exergame group BASFI scores (19).

## Discussion

Chronic low back pain as an entity is an immense societal cost and burden. Evidence-based treatment options are limited, despite decades of research on this subject. The standard of care for the treatment of CLBP currently is multi-modal therapy, and yet this is still largely ineffective (4–6). Virtual reality is a novel treatment option with burgeoning evidence and may be an option for the physician's armamentarium.

In this scoping study, we used wide inclusion criteria and yet only 13 published studies were identified. This is a clear representation of the fact that research in this area is nascent. Future studies are required to further analyze the nature of the effect and safety of treatment of CLBP with VR.

The findings of this scoping study, while perhaps not fully generalizable, do offer several important conclusions. First, published data demonstrates safety and tolerance of VR for patients with CLBP, and that use of VR in treatment of CLBP seems to be feasible. Second, limited data suggests that VR therapies can improve quality of life in patients with CLBP and pain associated with CLBP. These findings did not, however, translate to efficacy of functional improvement as there is conflicting data as to whether functional measures improve with VR treatment. This data is very heterogeneous and therefore cannot be combined meaningfully to make succinct recommendations. This heterogeneity is found in both the variety of treatments utilized and the study populations in question.

In summary, this scoping review outlines existing literature regarding VR and CLBP demonstrates that four themes currently exist in this literature: safety and feasibility, impact on quality of life, impact on pain, and impact on function. It is obvious that much more research is necessary before recommendations can be made for or against the use of virtual reality therapies in the treatment of chronic low back pain. As VR research continues to develop, investigators should continue to include measures of CLBP pain and health-related quality of life consistent with those used in prior studies. These measures demonstrated sensitivity to change in the present scoping review and adding to extant data should strengthen outcomes. Measures of physical function are not as robust, so more work is needed to determine whether VR holds promise for physical rehabilitation in patients with CLBP. Although the nascent research uncovered in this scoping review found good evidence for safety and tolerability of VR, more studies of safety, acceptance, and satisfaction are recommended including focused studies of spinal pain risks specific to use of VR. Overall, the methodological quality of studies reviewed in this scoping review was poor and outcomes were limited to short-term posttreatment outcomes. Future studies are strongly encouraged to improve scientific rigor and include long-term follow-up assessments to more precisely gauge the durability of beneficial VR outcomes.

## Author Contributions

All authors contributed in depth to the literature review, analysis of the manuscripts, writing of the manuscript, and editing of the manuscript.

## Conflict of Interest

The authors declare that the research was conducted in the absence of any commercial or financial relationships that could be construed as a potential conflict of interest.

## Publisher's Note

All claims expressed in this article are solely those of the authors and do not necessarily represent those of their affiliated organizations, or those of the publisher, the editors and the reviewers. Any product that may be evaluated in this article, or claim that may be made by its manufacturer, is not guaranteed or endorsed by the publisher.

## Abbreviations

6MWT: 6-Minute Walk Test
ACTH: Adrenocorticotropic hormone
ASQOL: Ankylosing Spondylitis Quality of Life Questionnaire
BASFI: The Bath Ankylosing Spondylitis Functional Index
BDI-II: Beck Depression Inventory-II
CPAQ-8: Chronic pain Acceptance Questionnaire
DVPRS: Defense and Veterans Pain Rating Scale
HOMA-IR: homeostatic model assessment insulin resistance
IKT: Isokinetic training
MPQ: McGill Pain Questionnaire
NPRS: numeric pain rating scale
ODI: Oswestry Disability Index
PCS: patient catastrophizing scale
PGIC: patient's global impression of change
PSEQ2: Pain Self-Efficiency Questionnaire
ROM: range of motion
RMDQ: Roland Morris Disability Questionnaire
SF36: (RAND corporation) Short form 36 item health survey
SUS: System Usability Scale
TEI: Treatment Evaluation Inventory
TUG: Timed-up and go test
VRT: virtual reality training.
